# Characterizing Social Insecurity in a Rural North Carolina Emergency Department

**DOI:** 10.5811/westjem.54605

**Published:** 2023-04-26

**Authors:** Elizabeth Gignac, Godwin Y. Dogbey, Aleksandr Pevtsov, Autumn Bass, Tibor Nagy, Amna Farshori, Grace D. Brannan

**Affiliations:** Campbell University School of Osteopathic Medicine, Harnett County, North Carolina

## Abstract

**Introduction:**

Social insecurity, a form of deprivation of social amenities, if present among patients presenting in a rural emergency department (ED) can be a source of medical burden and poor health outcomes. Although knowledge and understanding of the insecurity profile of such patients is necessary for targeted care that improves their health outcomes, the concept has not been comprehensively quantified. In this study we explored, characterized, and quantified the social insecurity profile of ED patients at a rural teaching hospital in southeastern North Carolina with a large Native American population.

**Methods:**

A paper survey questionnaire was administered by trained research assistants between May–June 2018 to patients who presented to the ED and consented to participate in this cross-sectional, single-center study. The survey was anonymous with no identifying information collected on the respondents. A general demographic section and questions derived from the literature capturing sub-constructs of social insecurity—communication access, access to transportation, housing insecurity and home environment, food insecurity, and exposure to violence–were captured in the survey. We assessed the factors included in the index of social insecurity based on a rank ordering using the magnitude of their coefficient of variation and the Cronbach’s alpha reliability index of the constituent items.

**Results:**

Overall, we collected 312 surveys from the approximately 445 administered and included them in the analysis, representing a response rate of about 70%. The average age of the 312 respondents was 45.1 (±17.7) years with a range of 18.0–96.0. More females (54.2%) than males participated in the survey. Native Americans (34.3%), Blacks (33.7%), and Whites (27.6%) comprised the three major racial/ethnicity groups of the sample, which are representative of the study area’s population distribution. Social insecurity was observed among this population regarding all the subdomains and an overall measure (P <.001). We identified three key determinants of social insecurity—food insecurity, transportation insecurity, and exposure to violence. Social insecurity significantly differed overall and among the three of its key constituent domains by patients’ race/ethnicity and gender (P <.05).

**Conclusion:**

Emergency department visits in a rural North Carolina teaching hospital are characterized by a diverse patient population, including patients with some degree of social insecurity. Historically marginalized and minoritized groups including Native Americans and Blacks demonstrated overall higher rates of social insecurity and higher indexes on exposure to violence than their White counterparts. Such patients struggle with basic needs such as food, transportation, and safety. As social factors play a critical role in health outcomes, supporting the social well-being of a historically marginalized and minoritized rural community would likely help build the foundation for safe livelihood with improved and sustainable health outcomes. The need for a more valid and psychometrically desirable measurement tool of social insecurity among ED populations is compelling.

## INTRODUCTION

Emergency departments (ED) across the United States (U.S.) frequently serve as medical safety nets for marginalized and excluded populations. The ED has become the oasis of primary healthcare access for patients who are uninsured, underinsured, low income, and homeless.^1^–^6^ Prior studies suggest that any forms of social deprivation can significantly and negatively impact health outcomes in a given population.^4^,^7^–^9^ The concept of social insecurity, in a health-related context, has been studied or described in various ways without a clear consensus. Studies involving any semblance of social insecurity have been situated within the context and conceptual framework of social determinants of health (SDOH).^4^,^7^,^10^,^11^

Social insecurity can be construed as the multitude of social factors that increase threats and risks to people’s lives and the likely negative impacts on their health outcomes.^10^ Social insecurity can be described as an overarching factor among the plethora of factors that underpins healthcare disparities in the U.S. It undergirds many of the variables associated with lack of access to affordable and quality healthcare.^7^ Underlying social insecurity is the coexistence of economic deprivation and inequity. Some researchers have measured a community’s degree of disparity using the Deprivation Index, which consists of four indicators: unemployment; social class; type of housing tenure; and car ownership.^7^, ^11^,^13^ Other variables such as race/ethnicity, income, food availability, and education are often incorporated in analyses of a community’s Deprivation Index.^11^,^12^

The literature on SDOH has highlighted the association between social factors and health outcomes of the population. However, studies that have coherently examined multiple factors in defining and characterizing social insecurity among rural populations have been scanty.^4^,^5^,^7^–^12^ Our study took a more coherent and comprehensive approach to explore, characterize, and quantify social insecurity in a unique and previously unstudied population. We explored the factors contributing to social insecurity in a rural community teaching hospital with a large Native American population. We hypothesized that patient demographics, namely age, race/ethnicity, and gender, would be associated with the key factors or sub-domains of social insecurity.

## METHODS

### Study Design and Location

This was a cross-sectional study conducted at the ED of University of North Carolina (UNC) Health Southeastern in Lumberton, NC. Lumberton is the most populated city in Robeson County, which is one of the largest and poorest counties in the state. Robeson County measures 973 square miles, and UNC Southeastern is the sole regional medical center in the county. This rural hospital serves a diverse, medically disinvested, and economically impoverished population. Life expectancy in Robeson County is the lowest of all counties in the state. In 2015 it ranked 100 of 100 counties in “health factors” and 95 of 100 counties in “health outcomes.”^6^,^21^–^24^ Additionally, Robeson County is home to the Lumbee Tribe of North Carolina, a state-recognized Native American tribe without federal benefits, which comprises nearly 40% of the population.^24^ Compared to the U.S. median household income of $63,179 during the study period, the median household income in Robeson County was just $34,976.^24^ Furthermore, only 14% of Robeson County residents have achieved an education level of Bachelor’s degree or higher, compared with nearly 33% of the U.S. population.^23^

**Population Health Research Capsule**

What do we already know about this issue?

*Social determinants of health critically impact the health outcomes of individuals and communitie*s.

What was the research question?

*We sought to determine, characterize, and quantify the social insecurity profile of a rural ED patient population*.

What was the major finding of the study?

*Significant race/ethnicity and gender differences exist between Native Americans/Blacks compared to Whites in three key constituent domains of social insecurity: Food insecurity, transportation insecurity, and exposure to violence. (P<.05)*.

How does this improve population health?

*Supporting the social well-being of historically marginalized rural populations is imperative for building safe and sustainable livelihoods with improved health outcomes*.

For this cross-sectional study, we implemented an intercept survey method with convenience sampling of ED patients at UNC Southeastern. Although it was a convenience sampling, it bore some resemblance to a quasi-random sampling. Research assistants (RA) were present at varying times in alternating sequence and, except for the exclusion criteria, every patient had equal opportunity to participate in the survey.

### Participants: Recruitment, Informed Consent, and Inclusion/Exclusion Criteria

The research team, including trained RAs, administered a survey questionnaire to ED patients who met inclusion criterion between May–June 2018. The inclusion criterion was subjects ≥18 years of age who completed a consent process. We excluded subjects who were non-English speaking, currently incarcerated, presented with psychiatric chief complaints, or those who presented as critically ill. Subjects were not screened for literacy, but upon a subject’s request, RAs provided verbal assistance with survey completion. The survey questionnaires were printed and placed in sealed envelopes by the subjects, and their anonymity was preserved.

### Construct of Social Insecurity

After reviewing prior research on SDOH, we identified five major domains as the framework for evaluating social insecurity.^1^,^5^,^7^,^13^ The five domains underlying the construct of social insecurity with their associated number of survey questions are as follows^9^:

- Communication access (3 items)
- Access to transportation (4 items)
- Housing security and home environment (3 items)
- Food insecurity (3 items)
- Exposure to violence (5 items)

The survey questions reflected these overarching themes found in various prior works involving SDOH. Additionally, we collected sociodemographic information from the participants. Beyond the five domains listed above, we aimed to identify other nuances of social insecurity.

### Data Analysis

We generated descriptive statistics, such as frequencies/percentages for categorical variables, and determined means, ranges, and standard deviations for continuous variables. To quantify social insecurity, we constructed a scoring index using items of the sub-domains and an overall score consisting of all the items together. We performed reliability analysis (as measured by the Cronbach’s alpha) for each item of the sub-domains, and for the overall construct of social insecurity. Furthermore, we used a rank ordering of the factors based on coefficient of variation (CV) in conjunction with the Cronbach’s alpha to select the factors to be included in the social insecurity index construction. We performed a preliminary multivariate analysis by using the sub-domains as dependent variables and demographics (age, gender, and ethnicity) as independent variables. Following that analysis, we determined statistically significant group differences with respect to continuous variables by using parametric (or non-parametric equivalents where necessary) tests such as analysis of variance (ANOVA), independent samples *t*-test, and one-sample *t*-test as appropriate. Statistical significance level was set at a *P*-value of of less than 5% for all inferential questions. We used SPSS Statistical Program version 27 (IBM Corporation, Armonk, NY) to analyze the data.

## RESULTS

### Demographics

The average age of the respondents was 45.1 (±17.7) years ranging from 18.0–96.0 years. Table 1 presents the demographic profile of the survey respondents. Overall, 312 surveys were collected from the approximately 445 administered and included in the analysis, representing a response rate of about 70%. Of the 312 respondents, 92.3% lived in Robeson County and 45.8% were male. The race/ethnicity distribution was almost evenly divided between Native American (34.4%), Black (33.7%), and White (27.6). The remainder was Hispanic or “other.” It should be noted that this demographic of race/ethnicity distribution of the survey participants/respondents intimately mirrors that of the population of Robeson County.^21^,^24^

### Domains and Item Analyses of Social Insecurity Manifestations

Table 2 outlines the response distribution over the five sub-domains of social insecurity among the respondent patients presenting to the ED of a large, rural teaching medical center. In all, 18 binary-anchored (0=no and 1=yes) items constituted social insecurity across each of the delineated five sub-domains. The items were intentionally calibrated such that a total response score of zero would indicate low while 18 would indicate high as a measure of overall social insecurity. We reverse coded items to correspond to the direction of the core items for consistency of the score—8 of the 18 items were reverse coded. The reverse coded items were 1, 2, 4, 5, 6, 7, 9, and 12 (refer to Table 2). For each of the sub-domains the ranges were 0–3 for communication, housing security and environment, food insecurity; 0–4 for transportation; and 0–5 for violence exposure. These ranges defined the number of items that composed each of the sub-domains of the overall social insecurity construct.

We conducted a reliability analysis on the items within each domain as well as for all the items overall. Table 3 shows the results of Cronbach’s alphas from the reliability analysis of the items in each sub-domain, as well as all the items together (overall). Three of the observed reliabilities and overall were fair and acceptable (Cronbach’s alpha ≥ 0.6); however, the negative reliability of the items underlying the communication sub-domain, although problematic, might hold plausible explanations that would be of policy relevance. Furthermore, the relatively low reliability for housing insecurity may need further exploration.

In Table 4, the mean of the overall summated social insecurity measure was higher than zero suggesting the presence of perceived social insecurity among the population under study. To account for scaling differences in the constituent number of items, rank ordering of the summated sub-domains from the highest to the least contributing subdomain to overall social insecurity (using CV = standard deviation/mean) yielded the following:

1. Housing insecurity
2. Food insecurity
3. Transportation
4. Exposure to violence
5. Communication

Although from the CV ordering, housing insecurity commanded a first place among the five sub-domains, its Cronbach’s alpha reliability index was not satisfactory: It was lower than the conventionally acceptable value of at least 0.6 for the purpose of this study. Hence, it would not be considered a factor in the quantification of the measurement of social insecurity in the population under study. Communication was the least in the CV ranking with even an unacceptable negative Cronbach’s alpha value. Thus, examining the results of the Cronbach’s alpha reliability analysis in tandem with the CV ordering, the top three factors of the five constituting social insecurity would be as follows: **exposure to violence**; **transportation**; and **food insecurity**—in relative increasing order of importance.

### Age, Gender, Race/Ethnicity, and Social Insecurity

In an initial multivariate analysis involving demographic variables age, gender, and race/ethnicity, age did not significantly predict any of the three social insecurity sub-domains. Following up using an ANOVA, we observed statistically significant differences among the three major ethnicity classifications of the population under study, namely Native American, Black, and White, regarding social insecurity overall and for each of the three key sub-domains—exposure to violence, transportation insecurity, and food insecurity. Native Americans/Alaska Natives and Blacks on average did not show a statistically significant difference in their index score of overall social insecurity (P=.79). However, Whites had on average, a statistically significant lower measure of social insecurity than American Indians/Alaska Natives (P< .001) and Blacks (P=.004). The results for the sub-domain of exposure to violence were similar; no statistically significant difference was observed between Native Americans/Alaska Natives and Blacks (P=1). However, statistically significant differences were observed between Whites and Native Americans/Alaska Natives (P<.001), and Blacks (P=.001). Whites on average had a lower index of exposure to violence than the other two race/ethnicity categories. On transportation insecurity, there was only a statistically significant difference between Native Americans/Alaska Natives and Whites (P=.03) with the former showing a higher index compared to the latter. Food insecurity yielded similar results, but in this case, the difference was between Whites and Blacks (P=.04).

Similarly, gender differences were observed for the overall measure of social insecurity as well as the three key sub-domains—exposure to violence, transportation, and food insecurity. Males on average than females exhibited higher overall social insecurity (P<.001). The same was true for exposure to violence (P<.001) and transportation insecurity (P=.004), but no statistically significant difference in food insecurity (P=.59) was observed.

## DISCUSSION

In this study we sought to determine, characterize, and quantify the most common elements of the social landscape that are associated with the patient population of this rural ED. The study presents an exploratory, descriptive, and quantitative characterization of social insecurity in a rural, underserved, and racially diverse county. Our results demonstrate higher levels of social insecurity among Native Americans/Alaska Natives and Blacks compared to White counterparts. This finding is consistent with numerous prior works that highlight the link between racial inequality and health outcomes in the US.^25^–^28^

Structural racism has been linked to poorer mental health, general health, and physical health through numerous pathways.^26^ Structural racism includes societal policies and systems that reinforce unequal access to housing, education, employment, credit, and healthcare. In turn, this can lead to poorer health outcomes, perpetuated discrimination, and unequal allocation of resources.^25^ Furthermore, racism contributes to poorer health outcomes by inflicting adverse cognitive and emotional stress, inducing allostatic and physiological stress, and potential physical injury from racially motivated assaults and violence.^25^, ^26^ Gleaning from our study, we found that Robeson County represents a microcosm of this national trend whereby structural and systemic racism may underpin the health-outcome discrepancies observed.^25^–^28^

Our results show that there was a significant level of social insecurity (although difficult to exactly benchmark or realistically quantify) among the study population. Of the five sub-domains of social insecurity delineated, three emerged as the most notable. Food insecurity was the topmost factor identified, followed by transportation availability, and exposure to violence.

Prior research suggests that rural residents of the western and southern US experience more food insecurity than their counterparts in other regions.^29^–^31^ Likewise, historically marginalized and minoritized populations incur higher rates of food insecurity than other groups.^29^–^31^ The rates of food insecurity in Robeson County were nearly twice the state average and more than double the national average.^29^ Although most respondents (88.1%) affirmed that they have access to healthy foods, a high proportion of them (27%) reported running out of food due to lack of money or reducing/skipping meals due to budget constraints. Interestingly, reported food insecurity was associated with higher rates of crime (ever having been robbed, threatened with a gun, or shot) than those without reported food insecurity.^32^

Transportation availability or access emerged as the second topmost source of social insecurity in this population. Subjects were surveyed on this topic to explore their ability to obtain outpatient specialty care when not available in Robeson County. As a rural, medically underserved county, many medical specialties are not available within Robeson County. It is not uncommon for ED patients to require outpatient follow-up with a specialist located at a distant urban area. Despite a high proportion (87.8%) reporting that they had reliable transportation within the county, over 30% did not have a valid driver’s license. Furthermore, 22.5% reported not having had reliable transportation to appointments, up to three hours away, outside the county. These findings highlight transportation barriers to obtaining healthcare within the population.

The third topmost factor contributing to social insecurity in the study population was exposure to violence. Our results suggest that many respondents had been victims of burglary and larceny: 28.5% reported a prior home robbery. In fact, the crime rate in Robeson County is consistently one of the highest in the country.^24^,^23^ Compared to urban Wake County, rates of violent crimes in Robeson County are nearly four times higher. Violent crime rates in Robeson County during the study dates were nearly triple the national rate.^33^–^34^

More than one in four respondents (26.9%) affirmed that they had been threatened with a gun, and about 10% reported personally sustaining a gunshot wound. Almost one third (31%) of respondents reported having a family member who had sustained a gunshot wound, and 17.4% reported having a family member die of a gunshot wound. Our results were consistent with prior works that have suggested a higher prevalence of gun violence among poor and minoritized populations than others. The implications of gun violence could be far-reaching. The sequelae of gun violence impact healthcare costs, disability, and mental health for victims and survivors.^18^–^24^,^32^–^34^

Housing insecurity and communication were found to be the least favorable factors, respectively, in the quantitative ranking of the five social insecurity domains examined in this study population. They did not yield basic, desirable, psychometric properties as sub-domains in the overall measure of the social insecurity construct. Nevertheless, they cannot be dismissed as unimportant factors in the SDOH framework. Further research may be warranted in quantifying their relative importance in a more coherent and comprehensive manner for development and measurement of the construct of social insecurity beyond that done in this study.

Although homelessness did not emerge as a top factor, many respondents in Robeson County had been affected by homelessness—an integral factor undergirding housing insecurity.^35^–^38^ With our finding of an affirmative response rate of 11.9%, it was suggestive that housing insecurity is more prevalent in Robeson County than other regions of NC. According to 2018 Continuum of Care data, the state of NC had approximately 8,962 homeless on any given day, representing a rate of 0.08% of a total state population of 10,383,620.^37^–^40^ Nationally, homelessness rates are reported to be 0.17%. It should be noted that our survey questions asked about homelessness over the prior year rather than currently.

Interestingly, our results suggest that homelessness is a possible risk factor for exposure to crime and gun violence. We observed that homeless respondents reported higher rates of home robbery and gun violence than those who did not report homelessness in the prior year. Of the homeless respondents, 48.6% affirmed that they had ever been robbed, 59.5% ever threatened with a gun, and 21.6% reported having ever been shot with a gun. These numbers were significantly higher on average than those reported in the general population. In fact, these rates were substantially higher than nationally reported rates of violence against homeless persons. The National Coalition for the Homeless reported that in 2016, for example, a total of 122 incidents of violent crime occurred among 578,424 homeless persons—a nationally reported, violent-crimes prevalence rate of 0.02%.^41^ This rate was in stark contrast to the 21.6% of homeless ED respondents in our study who reported having sustained a gunshot wound and 59.5% who had ever been threatened by a gun.

Surprisingly, the data suggests that communication by telephone, possessing a government-issued identification card, and having active utilities (water and electricity) in the home were not major challenges faced by the study population. In fact, 85.9% of respondents reported having a personal cell phone. A plausible explanation for the counterintuitive result could be that many respondents likely qualified for low-cost cell phone service such as Lifeline Support for Affordable Communities under a Federal Communications Commission assistance program. This program, at the study time, was available in all 50 states for people whose income level is at or below 135% of the federal poverty guidelines, and for those who qualified for other federal programs such as Medicaid, Supplemental Nutrition Assistance Program, or free public housing assistance.^42^–^44^

Despite high rates of personal cell phone ownership, 21.8% reported times when they did not have access to a phone if needed to make a phone call for health purposes. Notably, a high percentage of participants reported having a government-issued identification card (94.2%), which is required by many healthcare and social institutions. Furthermore, most respondents affirmed that they currently had running water and electricity in their home (98.7%). These results suggest that, although many residents of Robeson County live in poverty, most do not report deficiencies in access to phones and/or utilities.

## LIMITATIONS

Several limitations to this study should be noted. As a consented and convenience sampling survey, respondents may have been different from those who did not consent to participate. Consequently, a self-selection bias leading to more socially desirable responses was possible. Moreover, critically ill patients or those who presented with acute complaints were excluded. There was no way to force or coerce non-participants for any information, even their basic demographic information. Hence, we could only state this lack of comparison between participants and non-participants as a study limitation serving as a caution for the interpretation of the results.

Furthermore, participants were not screened for literacy. It is possible that a small number of illiterate participants did not seek verbal assistance from the RAs and provided unreliable responses. Moreover, the instrument used in this study did not demonstrate foolproof, desirable psychometric properties, and no general population sub-domain means existed for context of comparisons and benchmarking. Hence, it was difficult to benchmark a meaningful measure of “insecurity” exactly and realistically with established cut-off points. Additionally, non-English speaking patients were excluded from participation. According to US census data, 7.9% of households in Robeson County speak a language other than English in the home.^22^ It is plausible that non-English speaking status could be a factor associated with social insecurity, and this could be an area of future research.

While participants were not surveyed on income level or insurance status, race/ethnicity is a known factor associated with income levels and health insurance status/rates in the US.^40^–^41^ Prior works have noted large and pervasive differences over time in income by race/ethnicity, with Whites accruing higher incomes than Blacks, Hispanics, and Native Americans.^40^ Additionally, Whites have lower rates of uninsured persons compared to other racial/ethnic groups. The rates of uninsured non-Hispanic Blacks are nearly double the rate of uninsured Whites, and the rate of uninsured Hispanics is nearly four times higher than that of Whites.^41^,^43^ Demographic data from the study population, and Robeson County in general, suggests that poverty and lack of health insurance likely contribute to social insecurity in the study population. Finally, although the survey used in this study was similar to one used in a published study, neither was validated. In addition, the cross-sectional nature of the study regarding the data collected over a short period may have missed temporal variations that contribute to social insecurity.

Notwithstanding these limitations, this study could serve as a first step to rekindle the conversation about social insecurity among not only ED patients, but in patients throughout the healthcare system. Furthermore, it could provide the foundational framework for the development, construction, and quantification of a more valid measure of social insecurity in the US for rural, underserved populations that are similar to the current study population.

## CONCLUSION

This study highlights the social challenges facing ED patients in a rural North Carolina teaching hospital. Food insecurity, transportation difficulties, and exposure to violence stood as the top three of five factors of social insecurity studied. Historically marginalized and minoritized groups, including Native Americans and Blacks, demonstrated overall higher rates of social insecurity and higher indexes on exposure to violence than their White counterparts. Housing security and communication yielded perverse results that warrant further study.

Our findings suggest that multifaceted interventions targeted at violence reduction, easing transportation difficulties, and assuring food security are needed to improve the overall social well-being and health outcomes of Robeson County’s diverse, rural, and medically underserved population. Deliberate and targeted national policies addressing structural racism holistically would be necessary to improve socioeconomic outcomes, overall health, and well-being of individuals and communities, especially the historically marginalized. Finally, a more valid and robust measure of a comprehensively developed construct of social insecurity is warranted.

## Supplementary Information



## Figures and Tables

**Table 1:** Demographic characteristics of respondents (N=312).

Characteristic	Number of responses	Percent
Gender		
Female	169	54.2
Male	143	45.8
Race/Ethnicity		
Native American/Alaska Native	107	34.3
Black	105	33.7
Hispanic	3	1.0
White	86	27.6
Other (including more than one category)	11	3.5
Highest education completed		
Less than high school	75	24.0
High school graduate	130	41.7
Some college/associate degree	89	28.5
Bachelor’s degree	11	3.5
Advanced degree	7	2.2
Lives in Robeson County		
Yes	288	92.3
No	24	7.7

**Table 2:** Response frequencies by subdomains and associated items of social insecurity construct.

Subdomain and items		Yes		No	
	
		n	%	n	%
Communication					
1.	Do you have a traditional phone line (“land line”) in your home?	116	37.2	196	62.8
2.	Do you have a personal cell phone (not shared with another person)?	268	85.9	44	14.1
3.	Are there ever times you need to make a phone call, but do not have access to a phone?	68	21.9	242	78.1
Transportation					
4.	Do you have a government-issued identification card such as a driver’s license, state ID or passport?	294	94.5	17	5.5
5.	Do you have a valid driver’s license?	209	67.0	103	33.0
6.	Do you have reliable transportation to get to an appointment in Robeson County?	274	87.8	38	12.2
7.	Do you have reliable transportation to get to an appointment outside Robeson County? (For example, Raleigh, Durham, Chapel Hill, or Wilmington)	241	77.5	70	22.5
Housing security and home environment					
8.	At any time in the past 12 months have you been homeless?	37	11.9	274	88.1
9.	Does your home have running water and electricity?	308	98.7	4	1.3
10.	In the past 12 months have you been without water or electricity at home because the bill was not paid?	29	9.3	282	90.7
Food insecurity					
11.	Are there ever times when you run out of food because you do not have money to buy more?	85	27.2	227	72.8
12.	Do you have access to the types of food you believe are healthy?	273	88.1	37	11.9
13.	Do you ever have to cut the size of your meals or skip them because of limited budget for food?	84	27.1	226	72.4
Exposure to violence					
14.	Has your home ever been robbed?	89	28.7	221	71.3
15.	Have you ever been threatened with a gun?	83	26.9	226	73.1
16.	Have you ever been shot with a gun?	32	10.3	278	89.7
17.	Has anyone in your family ever been shot with a gun?	96	31.0	214	69.0
18.	Has anyone in your family ever died of a gunshot wound?	54	17.4	256	82.6

* Missing data was omitted; thus, n varies from item to item.

**Table 3:** Reliability coefficients for each subdomain and the overall.

Subdomain	Cronbach alpha
Communication	−0.147
Transportation	0.603
Housing insecurity	0.439
Food insecurity	0.713
Exposure to violence	0.627
Overall**	0.759

** Overall + mean summative score of the subdomains.

**Table 4:** Descriptive statistics of the subdomains and overall score of social insecurity.

Subdomain	N*	Minimum	Maximum	Mean#	SD	CV
Communication	312	0	3	0.99	0.69	0.70
Transportation	312	0	4	0.73	1.01	1.38
Housing insecurity	312	0	3	0.22	0.53	2.41
Food insecurity	312	0	3	0.66	0.98	1.48
Exposure to violence	310	0	5	1.14	1.31	1.15
Overall**	312	0	14	3.74	3.12	

* Sample size varied due to missing values.

** Due to missing values the range of the overall was 0–14 rather than the theorized 0–18.

# A one-sample t-test showed that all the means were different than zero, (P < 0.001), indicating somewhat the presence of social insecurity.

*CV*, coefficient of variation.
