# Supplementary material for: Characterizing Social Insecurity in a Rural North Carolina Emergency Department
Source: West J Emerg Med. 2023 Apr 26;24(3):538–46. doi: 10.5811/westjem.54605 (PMC10284507; doi:10.5811/westjem.54605)
Supplement: Supplementary file 1 [file wjem-24-538-s001.docx]

APPENDIX

Copy of Survey of Social Insecurity in a Rural North Carolina Emergency Department

**Patient Survey**

DEMOGRAPHICS

1. What is your age in years?

__________

2. Do you live in Robeson County?

Yes No

3. What is your gender?

Female Male

4. What is your ethnicity?

(1) American Indian/Alaska Native

(2) Asian

(3) Black/African American

(4) Hispanic/Latino

(5) Hawaiian/Pacific Islander

(6) White/Caucasian

(7) Other

5. Highest Education Completed

1. Less than High school
2. High school graduate
3. Some college or Associate’s degree
4. Bachelor’s degree
5. Advanced degree

COMMUNICATION

6. Do you have a traditional phone line (“land line”) in your home?

Yes No

7. Do you have a personal cell phone (not shared with another person)?

Yes No

8. Are there ever times you need to make a phone call, but do not have access to a phone?

Yes No

TRANSPORTATION

9. Do you have a government-issued identification card such as a driver’s license, state ID or passport?

Yes No

10. Do you have a valid driver’s license?

Yes No

11. Do you have reliable transportation to get to an appointment in Robeson County?

Yes No

12. Do you have reliable transportation to get to an appointment outside Robeson County? (For example, Raleigh, Durham, Chapel Hill or Wilmington)

Yes No

HOUSING

13. At any time in the past 12 months have you been homeless?

Yes No

14. Does your home have running water and electricity?

Yes No

15. In the past 6 months have you been without water or electricity at home because the bill was not paid?

Yes No

FOOD

16. Are there ever times when you run out of food because you do not have money to buy more?

Yes No

17. Do you have access to the types of food you believe are healthy?

Yes No

18. Do you ever have to cut the size of your meals or skip them because of limited budget (or doesn’t have the money) for food?

Yes No

SAFETY

19. Has your home ever been robbed?

Yes No

20. Have you ever been threatened with a gun?

Yes No

21. Have you ever been shot with a gun?

Yes No

22. Has anyone in your family ever been shot with a gun?

Yes No

23. Has anyone in your family ever died of a gunshot wound?

Yes No
